# Hyperglycaemia-induced methylglyoxal accumulation potentiates VEGF resistance of diabetic monocytes through the aberrant activation of tyrosine phosphatase SHP-2/SRC kinase signalling axis

**DOI:** 10.1038/s41598-018-33014-9

**Published:** 2018-10-02

**Authors:** Marc Dorenkamp, Jörg P. Müller, Kallipatti Sanjith Shanmuganathan, Henny Schulten, Nicolle Müller, Ivonne Löffler, Ulrich A. Müller, Gunter Wolf, Frank-D. Böhmer, Rinesh Godfrey, Johannes Waltenberger

**Affiliations:** 1 Experimental and Molecular Cardiology, Department of Cardiovascular Medicine, University Hospital Münster, Münster, Germany; 2 Institute of Molecular Cell Biology, Centre for Molecular Biomedicine, University Hospital Jena, Jena, Germany; 3 Department of Physiology, Cardiovascular Research Institute Maastricht (CARIM), Maastricht, The Netherlands; 4 Department of Internal Medicine III, University Hospital Jena, Jena, Germany; 5 Cells-in-Motion Cluster of Excellence (EXC 1003-CiM), University of Münster, Münster, Germany

## Abstract

Diabetes mellitus (DM) is a major cardiovascular risk factor contributing to cardiovascular complications by inducing vascular cell dysfunction. Monocyte dysfunction could contribute to impaired arteriogenesis response in DM patients. DM monocytes show blunted chemotactic responses to arteriogenic stimuli, a condition termed as vascular endothelial growth factor (VEGF) resistance. We hypothesize that methylglyoxal (MG), a glucose metabolite, induces monocyte dysfunction and aimed to elucidate the underlying molecular mechanisms. Human monocytes exposed to MG or monocytes from DM patients or mice (*db/db*) showed VEGF-resistance secondary to a pro-migratory phenotype. Mechanistically, DM conditions or MG exposure resulted in the upregulation of the expression of SHP-2 phosphatase. This led to the enhanced activity of SHP-2 and aided an interaction with SRC kinase. SHP-2 dephosphorylated the inhibitory phosphorylation site of SRC leading to its abnormal activation and phosphorylation of cytoskeletal protein, paxillin. We demonstrated that MG-induced molecular changes could be reversed by pharmacological inhibitors of SHP-2 and SRC and by genetic depletion of SHP-2. Finally, a SHP-2 inhibitor completely reversed the dysfunction of monocytes isolated from DM patients and *db/db* mice. In conclusion, we identified SHP-2 as a hitherto unknown target for improving monocyte function in diabetes. This opens novel perspectives for treating diabetic complications associated with impaired monocyte function.

## Introduction

Arteriogenesis is an important process to enhance blood flow in ischemic tissues by building functional arteries from pre-existing arterioles^1,2^. In patients with coronary artery disease, this process of collateral growth is necessary for the improvement of regional myocardial perfusion. However, arteriogenesis is impaired in diabetic patients compared to non-diabetic individuals^3–5^. Due to their poor collateral development, diabetic individuals are rather susceptible to cardiac events with respect to enhanced myocardial infarct size^6^ and elevated cardiovascular mortality^7^.

It has been established that monocytes are substantially involved in the arteriogenic process^8^ by producing growth factors^9^ and by remodelling of the media through metalloproteinases^1^. Monocytes have the capability to respond to arteriogenic stimuli and to home towards sites of vascular growth. This ability requires a chemotactic response of monocytes to arteriogenic stimuli like vascular endothelial growth factor A (VEGF-A) or the placental growth factor 1 (PlGF-1) defined as target-oriented migration. Both are ligands for vascular endothelial growth factor receptor 1 (VEGFR-1) capable of inducing VEGFR1 signalling mediating monocyte migration^10,11^. Primary human monocytes isolated from DM individuals exhibit a dysfunctional phenotype primarily characterized by a blunted chemotactic response to VEGFR1 agonists like VEGF-A and PlGF-1, previously described as “VEGF resistance”^12,13^.

The diabetic milieu, which contributes to cell dysfunction, consists of several components. One important component is methylglyoxal (MG), a highly reactive dicarbonyl^14^ which accumulates in DM patients. MG can form advanced glycation end products (AGE) and can induce oxidative stress^15^. Under diabetes-induced hyperglycaemic conditions the rate of glycolysis is elevated resulting in the increased production of dihydroxyacetone phosphate and glyceraldehyde-3-phosphate which then gets fragmented to MG^16^. Consistent with this notion, erythrocytes of diabetic patients showed 15- to 25-fold enhanced levels of MG compared to healthy individuals. The elevated levels of MG were considered to be sufficient for driving diabetes-related complications even in the absence of elevated blood glucose levels^17^. Several studies have shown that MG is accountable for various complications in DM, e.g. hyperalgesia in diabetic neuropathy^18^, enhanced atherogenicity through endothelial dysfunction^16^, reduced insulin sensitivity^19^, development of hypertension^20^ and decelerated wound healing^21^. Interestingly, a recent study reported a MG-mediated alteration in the VEGFR signal transduction pathway. MG induced a pathologically-relevant, accelerated angiogenesis phenotype in zebrafish embryos due to the enhanced activation of the VEGF receptor 2 (VEGFR-2)^22^.

SH2 domain-containing tyrosine phosphatase 2 (SHP-2/PTPN11) is a ubiquitously expressed cytosolic protein tyrosine phosphatase (PTP). SHP-2 has a similar overall structure and high homology with the hematopoietic cell-specific-SHP-1 phosphatase and is involved in the regulation of growth factor and cytokine signalling^23^. SHP-2 can be found in all mammals and mutations in the PTPN11 gene account for genetic diseases affecting the cardiovascular system, e.g. Noonan and LEOPARD syndrome^24^. Moreover, SHP-2 has been established as a relevant target in oncology since it is involved in different cancer types^25^. Recently, SHP-2 has been characterized as the responsible phosphatase mediating the enhanced motility of triple negative breast cancer cells by regulating SRC family kinases^26^. Furthermore, it has also been shown recently that SHP-2 positively mediates inflammatory responses in systemic lupus erythematosus (SLE). In addition, the inhibition of SHP-2 activity ameliorated SLE pathogenesis^27^.One of the primary targets of SHP-2 is SRC family kinases (SFK). SHP-2 is capable of dephosphorylating the Tyr-527-site of the SFK and thereby activate these kinases^28^ since phosphorylated Tyr-527 suppresses the kinase activity of SRC^29^.

Restoration of monocyte function in diabetic patients could be a viable therapeutic strategy to improve arteriogenic responses. However, the molecular determinants mediating monocyte dysfunction in diabetes are not well characterized and we aimed to understand the underlying mechanisms. To our knowledge, this is the first study in which the specific role of MG and its potential contribution in inducing monocyte dysfunction in DM has been investigated.

## Results

### Clinical characteristics

The clinical and biochemical characteristics of the individuals are presented in Table 1. All the DM patients included in the study were diagnosed with T2 DM with elevated levels of both blood glucose and HbA1c.

**Table 1:** Clinical characteristics of the non-diabetic and diabetic individuals.

	Non-DM	T2 DM	significance
N	24	33	
Age (years)	55.5 ± 14.8	64.6 ± 8.4	n.s.
Sex (male/female)	18/6	19/14	n.s.
BMI (kg/m^2^)	26.8 ± 3.9	34.9 ± 7.2	<0.001
HbA1c (%)	5.32 ± 0.65	7.98 ± 1.02	<0.05
HbA1C (mmol/mol)	34.65 ± 7.13	63.79 ± 11.14	<0.05
Glucose (mmol/L)	5.35 ± 0.85	11.27 ± 4.23	<0.05
Smoking (yes/no)	2/22	1/32	n.s.
Hypercholesterolaemia (yes/no)	7/17	19/14	n.s.

### The glucose metabolite MG imparts a pro-migratory and anti-chemotactic phenotype to monocytes

**Figure 1:**
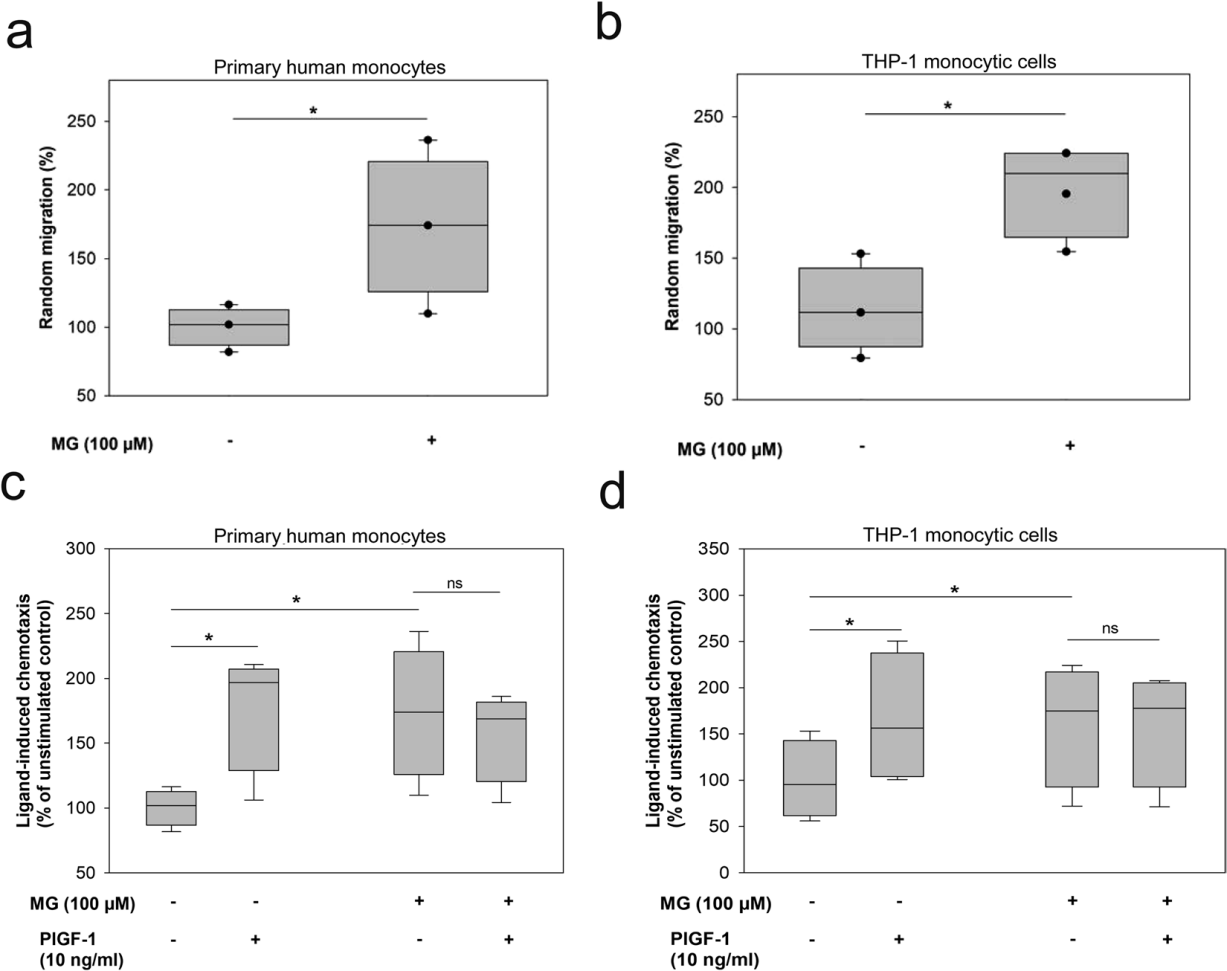
The glucose metabolite MG imparts a pro-migratory and anti-chemotactic phenotype to monocytes. Bars indicate counted monocytes on the membrane in percentage in relation to control (median set to 100%). (**a**) Primary human monocytes chemokinesis. Monocytes were incubated without or with 100 µM MG for 24 hrs. (**b**) Monocytic THP-1-cell line chemokinesis. Monocytes were incubated without or with 100 µM MG for 24 hrs. (**c**) Primary human monocytes chemotaxis. Monocytes were stimulated without or with 10 ng/ml PLGF-1. (**d**) Monocytic THP-1-cell line chemotaxis. Monocytes were stimulated without or with 10 ng/ml PLGF-1. Two sample independent t-test was used for statistical analysis (*P < 0.05, **P < 0.01).

First, we investigated the influence of MG on monocyte migration. Incubation of monocytes with 100 µM MG for 24 hrs significantly potentiated random migration (chemokinesis) by 73 ± 19.9% (p = 0.0125) in primary human monocytes (Fig. 1a) and 62 ± 20.2% (p = 0.0104) in the monocytic cell line THP-1 (Fig. 1b) in comparison to non-treated cells. Furthermore, directional migration (chemotaxis) of monocytes towards the arteriogenic stimuli, PlGF-1 (10 ng/ml)-a specific ligand for VEGFR-1, was examined. Primary human monocytes not incubated with MG showed an enhanced migration (71 ± 19.4%) following PlGF-1 (10 ng/ml) stimulation. Following 24 hrs incubation with 100 µM MG, primary human monocytes were refractory to PlGF-1 stimulation, i.e., stimulation with PlGF-1 (10 ng/ml) could not further increase directional migration of MG-exposed cells (Fig. 1c). Similarly, these findings could be recapitulated in the monocytic THP-1 cell line. Untreated cells responded towards PlGF-1 with 66 ± 8.2% enhancement in directional migration. Conversely, after incubation with MG, THP-1 cells were unable to respond to PlGF-1 stimulation (Fig. 1d). Furthermore, we could also detect impaired chemotactic response of MG-treated monocytes towards another VEGFR-1 agonist, VEGF-A. As shown in Supplementary Fig. S1, MG-treated monocytes displayed an impaired chemotactic response indicating that both VEGFR-1 ligands were unable to induce monocyte migration. Since PlGF-1 is a very specific and stronger ligand for activating VEGFR-1, we used PlGF-1 for the activation of VEGFR-1 in this study. In addition, to rule out the potential endotoxin contamination of MG, we have analysed the endotoxin levels in the MG preparation used for the experiments. As shown in Supplementary Fig. S2, we could not detect any significant levels of endotoxin in different concentrations (10, 30, 100 and 300 µM) of MG used for the experiments. Taken together, these findings indicate that the glucose-derived metabolite MG is able to induce VEGF resistance in human monocytes.

### Tyrosine phosphatase SHP-2 and its downstream target SRC is activated in MG-exposed monocytes

**Figure 2:**
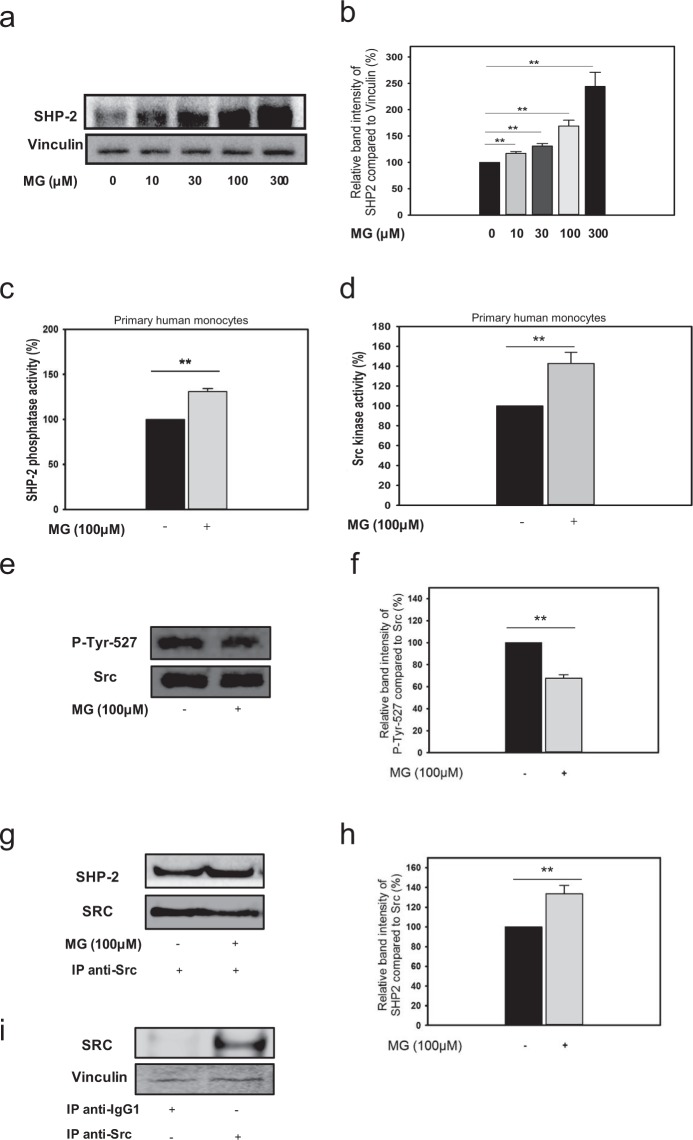
Tyrosine phosphatase SHP-2 and its downstream target SRC is activated in MG-exposed monocytes. Monocytes were incubated without (black bar) or with (grey bar) 100 µM MG for 24 hrs. The group with unexposed monocytes was set to 100%. Two sample independent t-test was used for statistical analysis (*P < 0.05, **P < 0.01). (**a**) Representative blots of SHP-2 expression at different concentrations of MG in relation to Vinculin. (**b**) Bar graph indicates quantified result in percentage. n = 3. (**c**) SHP-2 activity level, measured as released phosphatase from a synthetic phosphopeptide by SHP-2 immunoprecipitates (absorbance at 620 nm), in monocytes exposed to MG given as percentage. n = 4. (**d**) SRC activity level, measured as the extent of phosphorylation of a synthetic peptide by immunoprecipitated Src (absorbance at 450 nm) given as percentage. n = 7. Mann-Whitney Rank Sum Test was used to test statistical significance (*P = 0.05). (**e**) Representative blots of Phospho-SRC (Tyr527) expression in relation to SRC. (**f**) Bar graph displays result in percentage. n = 3. (**g**) Representative blot showing the co-immunoprecipitated SHP-2 in SRC immunoprecipitates. (**h**) Bar graph represents quantified result in percentage, n = 3. (**i**) Representative blot of control experiment with IP against IgG1-antibody.

Since SHP-2 was recently implicated in imparting a pro-motility phenotype to breast cancer cells^26^, we hypothesized that MG-induced random migration of monocytes could be mediated by SHP-2. Interestingly, MG exposed primary human monocytes showed significantly elevated SHP-2 expression levels in a dose dependent-manner compared to unexposed monocytes. 300 µM MG induced SHP-2 to the highest level (144 ± 27.0%) whereas lower concentrations of MG (10, 30, 100 µM MG) also induced SHP-2 expression at 17%, 31% and 69% respectively (Fig. 2a,b). Furthermore, monocytes incubated with MG exhibited significantly (p = 0.0021) augmented SHP-2 activity (31 ± 3.1%) when compared to the control group (Fig. 2c). Since the activity assays were performed under anaerobic conditions, we have ruled out the possibility of exogenous SHP-2 oxidation during cell lysis and subsequent processing. To test whether monocytes exposed to MG display an elevated SRC kinase activity, we tested the kinase activity of Src immunoprecipitates in MG-treated and untreated monocytes. In line with our hypothesis, we detected an elevated SRC activity (43 ± 11.8%, p = 0.0043) in MG-treated monocytes (Fig. 2d). In addition, we analysed the phosphorylation level of the downstream target of SHP-2. The Tyr-527-site of the SRC family kinases is highly phosphorylated at a steady-state level. Monocytes exposed to MG revealed significantly (32 ± 5.2%, p = 0.0018) lower levels of phosphorylated Tyr-527-SRC compared to untreated ones (Fig. 2e,f). Additionally, we investigated the modulation of the physical association between SHP-2 and SRC induced by MG-treatment. Co-immunoprecipitation of SHP2 with SRC clearly revealed a significantly (33 ± 8.7%, p = 0.0095) elevated SHP-2 association with SRC when the monocytes were exposed to MG (Fig. 2g,h) indicating that SHP-2 may directly interact with SRC and dephosphorylate the Tyr-527 site. The samples immunoprecipitated with IgG1 antibody (isotype control) revealed the specificity of the antibody (Fig. 2i).

### Deregulation of SHP-2 is a critical component in MG-induced monocyte dysfunction

**Figure 3:**
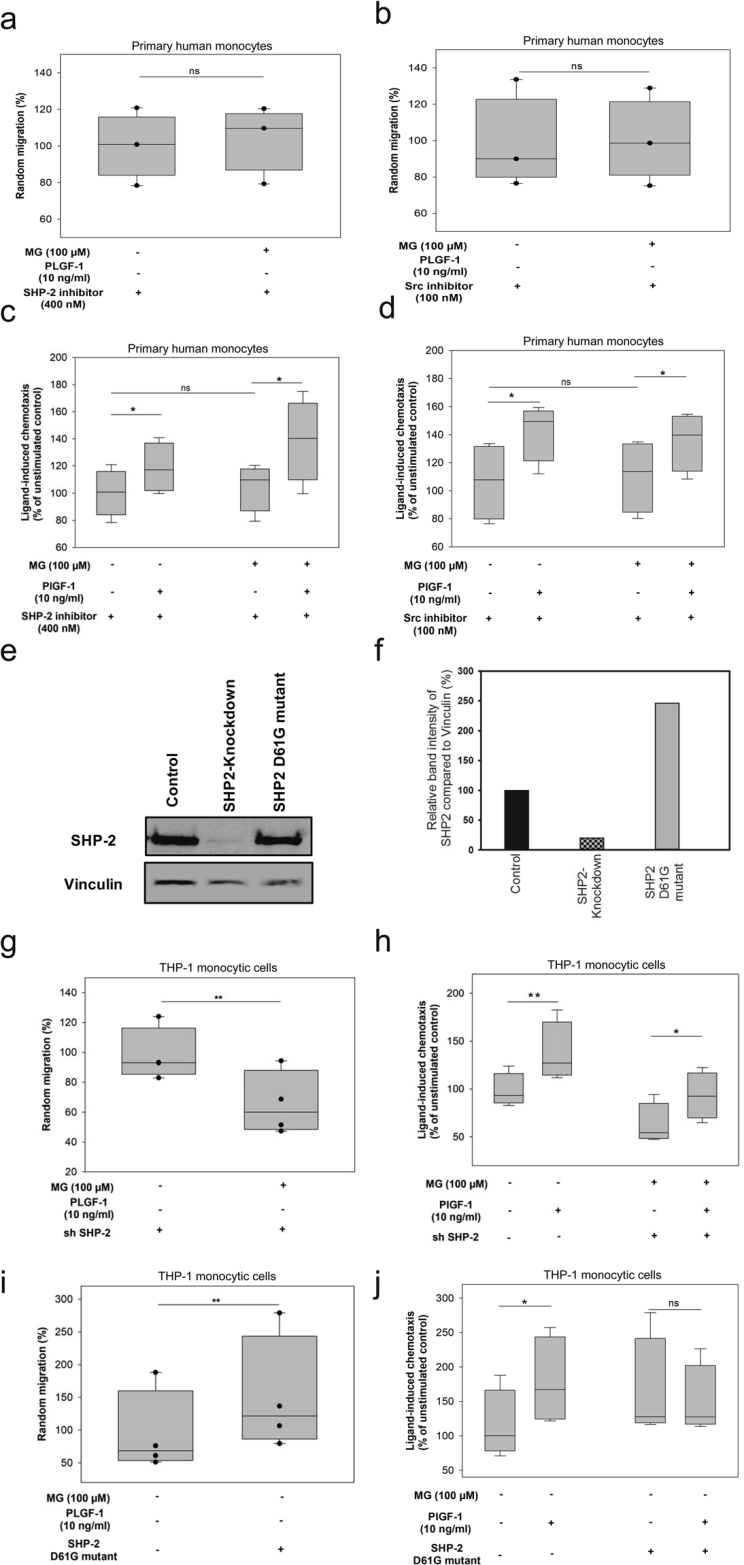
Deregulation of SHP-2 is a critical component in MG-induced monocyte dysfunction. For migration data **(a**–**d**, **g**–**j**) bars display counted monocytes on the membrane in percentage in relation to control (median set to 100%). Two sample independent t-test was used for statistical analysis (*P < 0.05, **P < 0.01, ***P < 0.001). (**a**,**b**) Monocyte chemokinesis after incubation with 400 nM SHP-2-inhibitor for 24 hrs or 100 nm SRC-inhibitor for 6 hrs. n = 3, respectively. (**c**,**d**) Monocyte chemotaxis after incubation with 400 nM SHP-2-inhibitor for 24 hrs or 100 nM SRC-inhibitor for 6 hrs. n = 3, respectively. (**e**) Representative blot of SHP-2-expression in genetically modified cells. (**f**) Bar graphic depicts quantified relative expression (control is set to 100%). (**g**) Random migration (chemokinesis) and (**h**) chemotaxis data of monocytes with knockdown of SHP-2 versus control. n = 3, respectively. (**i**) Random migration (chemokinesis) and (**j**) chemotaxis data of monocytes with constitutively active (D61G) mutant of SHP-2 versus control. n = 3, respectively.

To further challenge our hypothesis whether the activation of the SHP-2-SRC signalling axis is mediating MG-induced random migration (chemokinesis) of monocytes, we first inhibited this pathway pharmacologically. Application of 400 nM SHP-2-inhibitor alone was sufficient to block MG-impact on random migration. Both MG-exposed and unexposed monocytes revealed no significant difference in the level of chemokinesis when SHP-2 was inhibited (Fig. 3a). In line with this observation, in the presence of 100 nM SRC-inhibitor, MG treatment failed to induce chemokinesis in monocytes (Fig. 3b). Furthermore, use of either the SHP-2 or the SRC-inhibitor restored chemotactic response of the monocytes towards the arteriogenic stimulus PlGF-1 in the presence of MG. After PlGF-1 stimulation, the directional migration of monocytes treated with SHP-2-inhibitor was elevated by 35 ± 5.2% when compared to unstimulated MG-exposed monocytes (Fig. 3c). In addition, we could also reverse the refractoriness of MG-treated monocytes towards VEGF-A. As shown in Supplementary Fig. S3, SHP-2 inhibitor could improve the responses of MG-treated monocytes by 55 ± 10.2% compared to control. Similarly, SRC-inhibitor-treated monocytes responded to PlGF-1 and showed an elevated chemotaxis by 20 ± 13.0% (Fig. 3d). Additionally, to corroborate the findings obtained with pharmacological inhibitors, we employed genetically modified monocytic THP-1 cell lines with either a genetic depletion of SHP-2 or expression of constitutively active (D61G) SHP-2 (Fig. 3e,f). As shown in Supplementary Fig. S4, THP-1 monocytic cells with a stable SHP-2 knockdown or expressing constitutively active SHP-2 did not reveal any differences in their proliferative capacity when compared to their respective controls. When exposed to MG, monocytic cells with a SHP-2 knockdown (sh SHP-2) exhibited a significantly (p = 0.0036) lower level of chemokinesis (−36 ± 12.6%) compared to monocytes expressing normal SHP-2 levels (sh control) (Fig. 3g). Indeed, similar to what was observed with pharmacological inhibition of SHP-2, the inhibition of SHP-2 function using knockdown strategies partially reversed MG-induced monocyte dysfunction as seen by the efficient responses (18 ± 11.7%) towards arteriogenic stimuli, PlGF-1 (Fig. 3h). Interestingly, in contrast to what has been observed with SHP-2 knockdown, monocytic cells expressing the constitutively active gain of function mutant (D61G)^30^ of SHP-2 exhibited enhanced chemokinesis (55 ± 13.7%) compared to the control group (Fig. 3i). Furthermore, the D61G mutant cells, even in the absence of MG, displayed a blunted chemotactic response to arteriogenic stimuli, PlGF-1 (Fig. 3j). Taken together, these data demonstrate that SHP-2 contributes to aberrant monocyte function induced by MG.

### SHP-2 regulates the MG-induced activation of cytoskeleton protein paxillin through SRC kinase

**Figure 4:**
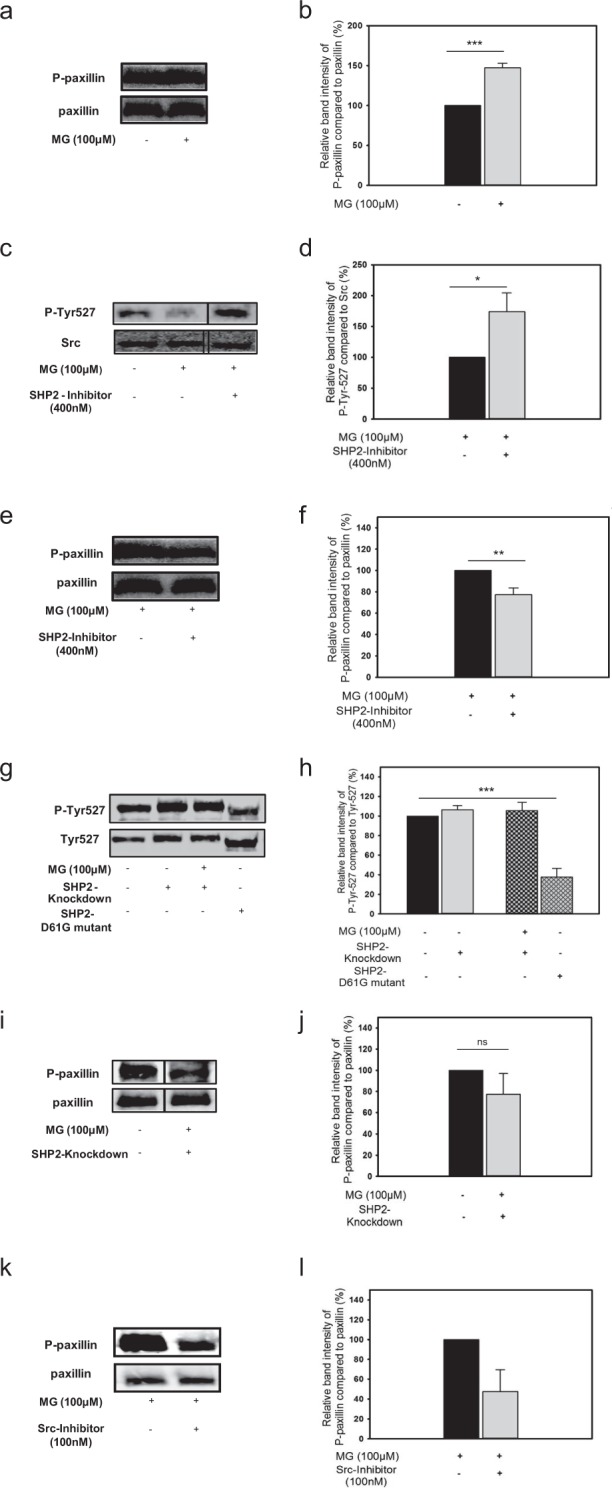
SHP-2 regulates the MG-induced activation of cytoskeleton protein paxillin through SRC kinase. Two sample independent t-test was used for statistical analysis (*P < 0.05, **P < 0.01, ***P < 0.001). (**a**) Representative blots and (**b**) corresponding quantification. Expression of phosphorylated paxillin in relation to total-paxillin. Monocytes were incubated without (black bar) or with MG (grey bar). n = 4. (**c**) Representative blots. Bands have been taken from different parts of the attendant blot. n = 3. (**d**) Corresponding quantification. Expression of phospho-SRC (Tyr527) in relation to SRC in MG-exposed monocytes without (black bar) or with (grey bar) SHP-2-inhibitor. n = 3. (**e**) Representative blots and (**f**) corresponding quantification. Expression of phosphorylated paxillin in relation to total-paxillin in MG-exposed monocytes without (black bar) or in the presence of (grey bar) SHP-2-inhibitor. n = 4. (**g**) Representative blots and (**h**) corresponding quantification. Expression of phospho-SRC (Tyr527) in relation to SRC (Tyr527) in SHP-2-knockdown and SHP-2-overexpressing monocytes compared to control monocytes. n = 3. (**i**) Representative blots. Bands have been taken from different parts of the attendant blot. n = 3. (**j**) Corresponding quantification. Expression of phosphorylated paxillin in relation to total-paxillin in MG-exposed monocytes with a genetic depletion of SHP-2 in comparison to control monocytes. n = 3. (**k**) Representative blots and (**l**) corresponding quantification. Expression of phosphorylated paxillin in relation to total-paxillin in MG-exposed monocytes without (black bar) or with (grey bar) SRC-inhibitor. n = 3.

Activation of SHP-2-SRC signalling axis could drive cell migration through phosphorylation-dependent activation of paxillin^31^. Therefore, we studied the phosphorylation level of paxillin in monocytes in different settings of MG exposure and SHP-2 loss of function and gain of function strategies. Incubation of monocytes with 100 µM MG for 24 hrs led to significantly higher (47 ± 5.6%, p < 0.0001) phosphorylation of paxillin (Fig. 4a,b). Despite MG-exposure, the enhanced dephosphorylation of the inhibitory Tyr-527-site of the SRC family kinases (Fig. 2e,f) was abrogated in the presence of SHP-2-inhibitor, which resulted in enhanced phosphorylation (83 ± 24.3%) of the Tyr-527-site (Fig. 4c,d). If SHP-2 drives MG-induced paxillin phosphorylation, inhibition of SHP-2 function should reduce the activation of paxillin. As expected, inhibition of SHP-2 contributed to the reduced (−23 ± 6.4%) phosphorylation of paxillin (Fig. 4e,f). Concordantly, cells from the monocytic cell line THP-1 with a genetic depletion of SHP-2 were not susceptible to the molecular changes induced by MG. Both phosphorylation level of the Tyr-527-site (Fig. 4g,h) and the levels of phosphorylated paxillin (Fig. 4i,j) revealed no significant change due to MG-exposure. Of note, monocytes with a knockdown of SHP-2 even showed an opposing trend of decreased (23 ± 19.7%) phosphorylation of paxillin even in the presence of MG (Fig. 4i,j). Interestingly, cells expressing a gain of function mutant (D61G) could mimic the MG effects by suppressing the phosphorylation of Tyr-527 by 62 ± 8.7% (Fig. 4g,h). Furthermore, the MG-induced enhanced phosphorylation of paxillin could be reduced by 52 ± 22.0% by the pharmacological inhibition of SRC (Fig. 4k,l). All the results in this section indicate that MG-induced SHP-2 activation mediates monocyte chemokinesis through SRC kinase-induced phosphorylation of paxillin.

### SHP-2-dependent impairment of monocytes can be recapitulated in monocytes from diabetic patients and *db/db* mice

**Figure 5:**
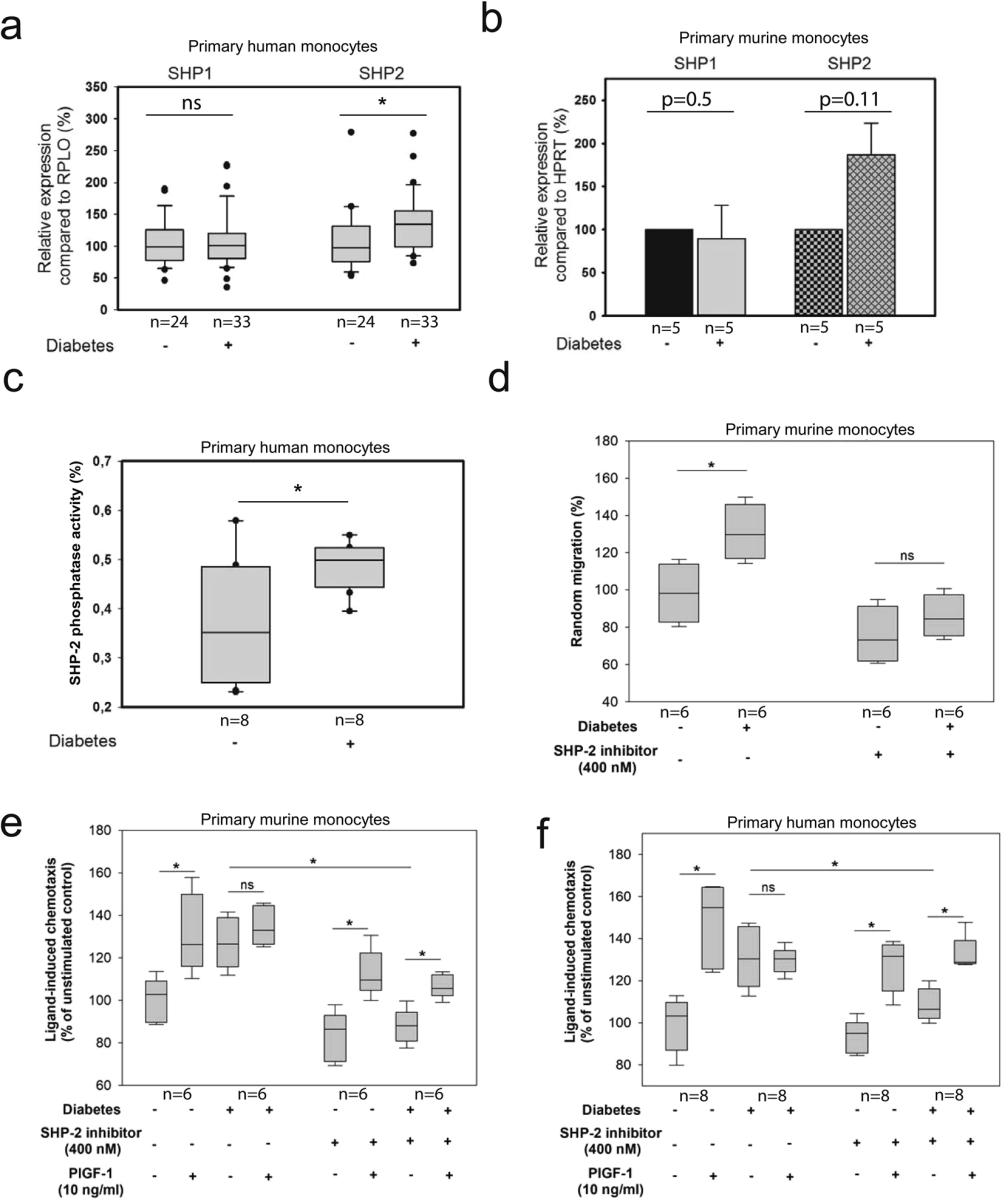
SHP-2-dependent impairment of monocytes can be recapitulated in the monocytes from diabetic patients and *db/db* mice. (**a**) SHP-1 and SHP-2 mRNA expression in monocytes from diabetic patients (n = 33) and healthy individuals (n = 24). Mann-Whitney Rank Sum Test was used to test statistical significance (*P < 0.05, **P < 0.01, ***P < 0.001). (**b**) SHP-1 and SHP-2 mRNA expression in monocytes from *db/db* mice (n = 5, grey) and Wt mice (n = 5, black). (**c**) SHP-2 phosphatase activity level, measured as released phosphatase from a synthetic phosphopeptide by SHP-2 immunoprecipitates (absorbance at 620 nm), in monocytes from diabetic patients (n = 8) and healthy individuals (n = 8). Mann-Whitney Rank Sum Test was used to test statistical significance (*P = 0.05). (**d**) Monocyte chemokinesis analysis using non-diabetic (n = 6) and diabetic murine monocytes (n = 6) after incubation with 400 nM SHP-2-inhibitor. Two sample independent t-test was used for statistical analysis (*P < 0.05). Bars display counted monocytes on membrane in percentage in relation to control (median set to 100%). (**e**) Analysis of monocyte chemotaxis towards PlGF-1 using non-diabetic (n = 6) and diabetic murine monocytes (n = 6) after incubation with 400 nM SHP-2-inhibitor. Kruskal-Wallis One Way Analysis of Variance on Ranks with Tukey correction was used for statistical analysis (*P < 0.05). Bars display counted monocytes on membrane in percentage in relation to control (median set to 100%). (**f**) Analysis of monocyte chemotaxis towards PlGF-1 using non-diabetic (n = 8) and diabetic human monocytes (n = 8) after incubation with 400 nM SHP-2-inhibitor. Kruskal-Wallis One Way Analysis of Variance on Ranks with Tukey correction was used for statistical analysis (*P < 0.05). Bars display counted monocytes on membrane in percentage in relation to control (median set to 100%).

To investigate whether monocytes from patients suffering from diabetes mellitus exhibit an enhanced expression of SHP-2, we isolated primary human monocytes of venous blood from both healthy individuals (n = 24) and diabetic patients (n = 33). The mRNA expression analysis using RT-qPCR indicates a significantly (28 ± 5.8%, p = 0.0325) augmented SHP-2 mRNA expression in diabetic monocytes compared to the control group (Fig. 5a). The expression of a structurally similar phosphatase, SHP-1, remained unaltered (Fig. 5a). Additionally, analysis of the SHP-2 expression in murine monocytes isolated from *db/db* mice (n = 5) and Wt (n = 5) controls revealed a similar trend (87 ± 37.2%) (Fig. 5b). Importantly, the SHP-2 activity of monocytes from DM-patients (n = 8) was found to be elevated (30 ± 4.8%), confirming that upregulation of SHP-2 activity is a predominant feature in DM-patient-derived monocytes (Fig. 5c). Furthermore, monocytes isolated from *db/db* mice recapitulated the exact phenotype of monocytes subjected to MG. These cells displayed an enhanced (28 ± 2.6%) chemokinesis compared to monocytes taken from the Wt mice. Of particular importance is the finding that pharmacological inhibition of SHP-2 could suppress the level of chemokinesis of diabetic monocytes (from *db/db* mice, n = 6) to a level similar to the chemokinesis displayed by non-diabetic monocytes (from Wt mice, n = 6) (Fig. 5d). Furthermore, monocytes from *db/db* mice (n = 6) and DM patients (n = 8) did not respond significantly towards PlGF-1 and this impaired responsiveness towards PlGF-1 could be restored completely by the use of SHP-2-inhibitor indicating clearly that therapeutic targeting of SHP-2 could reverse monocyte dysfunction (Fig. 5e,f). Since the application of SHP-2 inhibitor showed a tendency towards reducing the general migratory potential of monocytes, we carried out experiments with two different concentrations of SHP-2 inhibitor and analysed the basal and PlGF-1-induced migratory responses of monocytes. As shown in Supplementary Fig. S5, the application of SHP-2 inhibitor did not significantly alter the basal or PlGF-1-induced migration of cells compared to DSMO control. Taken together, we show that the impaired monocyte function induced by MG could be recapitulated in monocytes obtained from T2 diabetic human and *db/db* murine samples and SHP-2 inhibition *ex vivo* could reverse this monocyte dysfunction.

## Discussion

Our study identifies MG as an important glucose metabolite, which can independently alter the function of monocytes and leads to the development of VEGF resistance in monocytes. This is substantiated by the results presented in this study showing that we could induce the previously described VEGF resistance in human diabetic monocytes^11,12^ by incubating the monocytes with MG alone. This inability of monocytes to respond to PlGF-1, the specific growth factor which activates VEGFR1, represents the monocyte dysfunction/VEGF resistance phenotype. In our study, monocytes exposed to MG showed an upregulation and activation of SHP-2 which subsequently led to the dephosphorylation of the inhibitory tyrosine phosphorylation site 527 in SRC kinase. Subsequently, the activated SRC kinase phosphorylated its downstream target, paxillin, and this correlated to an increase in the non-directional migration of these cells. This non-specific activation of monocytes blunted their ability to respond to and migrate towards arteriogenic stimuli like PlGF-1. Even though it is known that MG is a potent inducer of vascular dysfunction^32^, most of the studies focussed on the effects of MG on endothelial cells and the potential alterations on mononuclear cell function remains partially characterized. To the best of our knowledge, this is the first study in which MG-induced alterations in cellular signalling lead to impaired monocyte function.

Our results are consistent with the observation that monocyte function is heavily compromised in diabetic patients^12,13^. MG is known to be abundantly present in diabetic patients^17^. DM-conditions are known to induce apoptosis of mononuclear cells resulting in the compromised cell function^33^. Since monocytes are critically involved in the collateral remodelling process^8^, any compromise in monocyte function could result in an impaired ability to develop arteries from pre-existing arterioles. Our data pinpoint the significant role played by MG as an independent factor in inducing monocyte dysfunction. Our results argue that elevated MG levels in diabetes could directly compromise monocyte-dependent vascular repair processes like arteriogenesis^3–5^ and result in an inferior outcome of diabetic patients suffering from cardiac events^7^.

**Figure 6:**
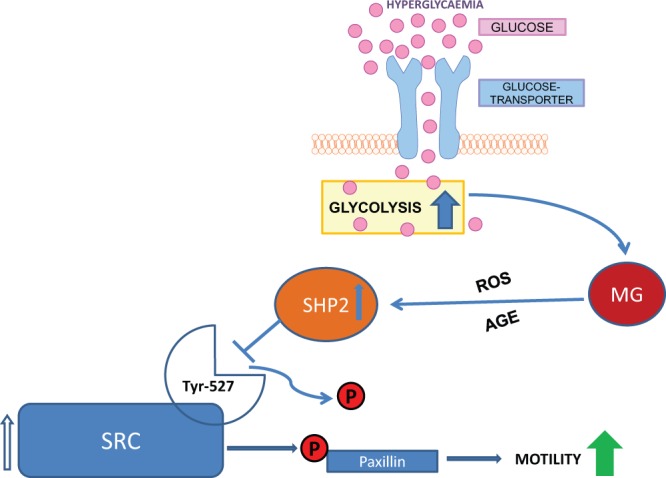
Model for MG-induced monocyte dysfunction. In diabetic patients, hyperglycaemic conditions are more frequent so that monocytes have an increased uptake of glucose. Therefore, the rate of glycolysis is elevated in monocytes resulting in the accumulation of MG. MG, either through enhanced AGE signalling or through ROS-induced inflammatory signalling possibly contributes to the transcriptional upregulation of SHP-2 expression and activity. This allows SHP-2 to physically interact with and efficiently dephosphorylate the inhibitory Tyr-527-site of the SRC family kinase. This results in the augmentation of SRC family kinase activity. Through the direct or FAK-mediated phosphorylation of paxillin, monocytes acquire an enhanced potential to randomly migrate. This heightened chemokinesis leads to an unresponsiveness of the monocytes for ligands of the VEGF-receptor, called VEGF-resistance.

Identification of SHP-2 as a molecular determinant which induces altered monocyte function is in line with perceptions gathered from several studies during recent years that SHP-2 is an important pathological factor contributing to various diseases. It is known that SHP-2 mutations drive genetic diseases^34^ and carcinosis, e.g. in breast cancer^26^, leukaemia^35^, lung cancer and neuroblastoma^36^. Notwithstanding, this is the first report pinpointing MG as a potent driver capable of inducing the activity and expression of SHP-2. Indeed, the specific mechanism behind this induction remains unclear. From published data, we could hypothesize that two different pathways could mediate MG-induced SHP-2 expression (Fig. 6). It is well known that MG is a precursor for the formation of AGEs^37^. Therefore, AGEs could mediate SHP-2 expression through the activation of AGE-receptor for AGEs (RAGE) signalling. Such a possibility has already been proposed to be operational in vascular smooth muscle cells^38^. On the other hand, MG promotes reactive oxygen species (ROS) production^15^ resulting in oxidative stress which could in turn activate transcription factors such as NFκB and AP1 which are known to bind to SHP-2 promoter and activate its transcription^38,39^. It is also possible that these two mechanisms contribute in a synergistic manner to elevated SHP-2 expression. Elucidating these interactions should be part of further studies.

SHP-2 is known to get oxidized and inactivated in environments with higher oxidative stress^40^. In addition, it is known that diabetes or MG accumulation induces ROS generation^15^ and could result in the functional inactivation of SHP-2. Interestingly, ROS accumulation and total phosphatase activity and the specific activity of PTP1B in diabetic monocytes are known to be inversely correlated^13^ (Godfrey *et al*., unpublished). However, we found that the specific activity of SHP-2 in MG-exposed cells was elevated. Furthermore, the finding that the DM-patient-derived monocytes displayed an enhanced SHP-2 activity further confirms that diabetic conditions could upregulate the enzymatic activity of SHP-2. This enhanced activity could be mostly due to the upregulation of SHP-2 expression as we have seen in MG-treated monocytes or monocytes isolated from DM-patients.

SHP-2 stimulates signalling pathways in opposition to many of the other PTPs^41^. Our observation that SHP-2 is able to activate SRC family kinases is consistent with published work^26^. It is known that SRC-paxillin interaction mediates the augmented motility of the triple-negative breast cancer tumor cells^26^ and that it is involved in the metastasis of neuroblastoma in children^42^. Our results clearly demonstrate that a similar mechanism is operating in diabetic monocytes due to MG accumulation. MG-treatment enhanced the physical interaction of SHP-2 with SRC and resulted in the dephosphorylation of the negative inhibitory tyrosine (pY 527 site) of SRC kinase. Even though Csk is known to mediate the effects of SHP-2 on SRC^28^, our results indicate that SHP-2 has a direct effect on SRC as evidenced by its direct interaction with SRC. However, further studies are required to understand the potential contribution of Csk in mediating SHP-2-induced effects in diabetic monocytes.

Activated SRC could directly modulate cell motility by the activation of cytoskeletal proteins like focal adhesion kinase (FAK) and paxillin^26^. In line with this observation, MG-induced monocytes displayed an enhanced  SRC kinase activity and we also detected an activation of paxillin in these cells. The activation of paxillin is completely blunted when the SHP-2 function is blocked. However, it is not clear whether SRC phosphorylates paxillin directly or indirectly through the FAK^42^.

Pharmacological inhibition of SHP-2 in monocytes from the *db/db* mice and DM patients clearly reversed the aberrant random motility and VEGF resistance phenotype. On the opposite, expression of a constitutively active (D61G) mutant of SHP-2 recapitulated VEGF resistance. Hence, we can emphasize the inhibition of SHP-2 as a novel strategy to relief VEGF-resistance in monocytes caused by MG. Interestingly, recent studies revealed the practical consideration of pharmacological inhibition of SHP-2 *in vivo*. This is demonstrated by the observation that mice with a knockdown, as opposed to a knockout of SHP-2^43^ are alive^44^ although SHP-2 is involved in several physiological cell processes^35,45^. Moreover, different recent reports on the usage of SHP-2 inhibitors indicate promising results as future therapeutic cancer drugs^46,47^ and as a potential drug to treat SLE^27^.

Recognizing the conjunction between SHP-2 and cancer together with our findings that SHP-2 is responsible for harmful consequences of diabetes mellitus, pharmacological SHP-2 inhibition could also act as a preventive therapy against the development of cancer in diabetic patients. This is a potentially interesting and controversial area about the association between diabetes mellitus and cancer^48^.

In summary, our present findings highlight a hitherto unknown mechanism by which monocytes are rendered dysfunctional in diabetic patients. Our work stresses the importance of undertaking further studies about SHP-2 and its role in contributing to various cardiovascular and diabetic pathologies.

## Materials and Methods

### Reagents

Methylglyoxal was from Sigma-Aldrich. SHP-2-inhibitor, NSC 87877 was from Tocris Bioscience, SRC-inhibitor-1 was from Santa Cruz Biotechnology. Protein tyrosine phosphatse activity assay kit was from Promega. Universal Tyrosine kinase assay kit was from Takara. RT-qPCR primers, hRPLO, hSHP-1, hSHP-2, mSHP-1, mSHP-2 were obtained from Sigma Aldrich.

### Clinical cohorts

Blood samples were obtained over a period of 18-months, from 57 individuals with type 2 diabetes (n = 33) or non-diabetic individuals (n = 24) visiting the department of Internal Medicine III, University Hospital Jena, Jena, Germany and the department of Cardiology, University Hospital Münster, Münster, Germany. Individuals with infections or with anti-inflammatory therapy were excluded.

### Ethics

This study was approved by the local ethics committee of Münster University Hospital, Germany and Jena University Hospital, Germany. All the DM patients and non-DM individuals provided written informed consent to participate in the study. The ethical permission number is 2011-612-f-S (Münster) and 4125-06/14 (Jena). The study conforms to the declaration of Helsinki.

### Isolation of Primary Human and Murine Monocytes

Monocytes were isolated from thrombocyte reduction filters obtained from venous blood of healthy individuals as previously described^13^ using Magnet assisted cell sorting (MACS) using negative selection. The Monocyte Isolation Kit II human from Miltenyi Biotec was used. For murine monocyte isolation, the bone marrow monocyte isolation kit from Miltenyi Biotec was used. Detailed protocol can be found in the electronic supplementary information.

### Animals

We used diabetic *db/db* (B6.Cg-Dock7m Leprdb/++/J) and non-diabetic Wt mice (Jackson Laboratory, Bar Harbor, ME) as controls. The animals were housed in a temperature-controlled environment and had free access to water and regular diet (65% carbohydrate, 11% fat, 24% protein). The animal experiments were carried out at the animal facility of the Cardiovascular Research Institute Maastricht (CARIM), Maastricht, the Netherlands. Ethical permission number is DEC#2014-046. All animal studies were in accordance with the German animal protection law and with the European Communities Council Directive 86/609/EEC and 2010/63/EU for the protection of animals used for experimental purposes. All experiments were approved by the Local Institutional Animal Care and Research Advisory Committee and permitted by the local authority. 8–12 weeks old mice were used. Bone marrow-derived monocytes were used for the experiments. n = 5–6 each for diabetic *db/db* mice and non-diabetic Wt mice.

### Cell culture and treatment

Primary human monocytes and the monocytic cell line THP-1 (acquired from Leibniz Institute DSMZ – German Collection of Microorganisms and Cell Cultures) were cultured in RPMI-1640 medium (+L-Glutamine, −D-Glucose, Thermo Scientific) supplemented with 5 mM Glucose, 25 mM Mannitol, 10% heat-inactivated fetal bovine serum (FBS) and 1% Penicillin/Streptomycin. For migration experiments and signalling studies cells were starved for 2 hrs in FBS free medium. Monocytes were kept in an incubator at 37 °C and 5% CO_2_. Wherever indicated, cells were incubated with 400 nM SHP-2-inhibitor for 24 hours (hrs) or 100 nM SRC-inhibitor-1 for 6 hrs. Cell cultures were tested for the absence of mycoplasma using the MycoFluor Mycoplasma Detection Kit (Thermo Scientific).

### Migration experiments

Monocyte chemokinesis and chemotaxis were studied with the modified 48-well Boyden chamber (Nucleopore) as previously described^11^. For quantification migrated cells were counted by 20 high power fields in four different wells using the Axioskop 2 Plus microscope (Carl Zeiss). Detailed protocol can be found in the electronic supplementary information.

### SHP-2 Phosphatase and SRC kinase activity measurement

Measurement of SHP-2 activity was done according to Godfrey *et al*.^49^, with minor modifications. Steps to measure SHP-2 activity were performed on ice and inside of an anoxic chamber (GB2202-P-V, MBraun). The activity of SHP-2 was measured in immunoprecipitates using the Tyrosine Phosphatase Assay System (Promega). Detailed protocol can be found in the electronic supplementary information.

### Western Blot and Immunoprecipitation

Immunoprecipitation was done as previously described^50^. A preclearing step was included which is described in “Phosphatase activity measurement”. For the detailed WB protocol, refer the electronic supplementary information. The primary antibodies used were anti-SHP2 (SC-7384), anti-Src (SC-18) from Santa Cruz Biotechnology (SCB), at a 1/1000 dilution, anti-Paxillin (2542), anti- pPaxillin (Tyr 118, 2541), anti-p527 Src (9935) from Cell Signalling Technology, at a 1/1000 dilution. The secondary antibodies were from SCB.

### Gene expression analysis

Detailed protocol and the sequences of the primers used can be found in the electronic supplementary information, Table S1. All the samples were run in duplicates and the results with a difference higher than 0.2 Ct between duplicates was not considered for analysis.

### Production of pseudoviral particles and THP-1 transduction

Production of retroviral particles for SHP-2 D61G cell generation in a SHP-2 knockdown background, lentiviral particles for SHP-2 knockdown and transduction of THP-1 cells was done as specified in Arora *et al*.^50^. Detailed protocol can be found in the electronic supplementary information.

### *Ex vivo* analysis of murine monocytes from diabetic *db/db* mice

Bone marrow cells from tibia and fibula were extracted by flushing with PBS-BSA solution. After erythrolysis, the cells were subjected to magnet-assisted cell sorting (MACS) according to the manufacturer’s instructions. The monocyte isolation kit from Miltenyi Biotech was used. The purity of isolated cells was confirmed by FACS and they were around 98% pure.

### Study design, blinding and statistical analysis

All the migration experiments were carried out with four technical replicates per condition. n = 3–8 were done for all experiments. Expression analysis of SHP-2 was carried out using monocytes from non-diabetic individuals (n = 24) and diabetic individuals (n = 33). Specific details about the design of the experiments are given in the figure legends. Animal experiments were blind to group assignment. All statistical analyses are presented as means ±SEM. The alpha level was 0.05. No statistical method was used to determine sample size. To analyse significance of differences in experiments with monocytes isolated from diabetic or healthy individuals/mice, the Mann-Whitney Rank Sum Test (for intergroup comparisons) or Kruskal-Wallis One Way Analysis of Variance on Ranks was used. For all the other experiments two sample independent t-test was performed, respectively. The exact test used is indicated in the figure legends. SigmaPlot software was used for statistical analysis. Level of significance was defined as P < 0.05.

## Electronic supplementary material

Supplementary Information

## Data Availability

The datasets used and/or analysed during the current study are available from the corresponding authors on reasonable request.
