# Supplementary material for: Hyperglycaemia-induced methylglyoxal accumulation potentiates VEGF resistance of diabetic monocytes through the aberrant activation of tyrosine phosphatase SHP-2/SRC kinase signalling axis
Source: Sci Rep. 2018 Oct 2;8:14684. doi: 10.1038/s41598-018-33014-9 (PMC6168515; doi:10.1038/s41598-018-33014-9)

Electronic Supplementary Information

Hyperglycaemia-induced methylglyoxal accumulation potentiates VEGF resistance of diabetic monocytes through the aberrant activation of tyrosine phosphatase SHP-2/SRC kinase signalling axis

Marc Dorenkamp[1,5], Jörg P. Müller[2], Kallipatti Sanjith Shanmuganathan[1,5], Henny Schulten[1,3], Nicolle Müller[4], Ivonne Löffler[4], Ulrich A. Müller[4], Gunter Wolf[4], Frank-D. Böhmer[2], Rinesh Godfrey*[1,3,5,6] and Johannes Waltenberger*[1,5,6]

[1]Experimental and Molecular Cardiology, Department of Cardiovascular Medicine, University Hospital Münster, Münster, Germany; [2]Institute of Molecular Cell Biology, Centre for Molecular Biomedicine, University Hospital Jena, Jena, Germany; [3]Department of Physiology, Cardiovascular Research Institute Maastricht (CARIM), The Netherlands; [4]Department of Internal Medicine III, University Hospital Jena, Jena, Germany; [5]Cells-in-Motion Cluster of Excellence (EXC 1003-CiM), University of Münster, Münster, Germany

[6]shared senior authorship

***Correspondence and requests for materials should be addressed to R.G or J.W:**

Dr. Rinesh Godfrey, PhD, Experimental and Molecular Cardiology, Department of Cardiovascular Medicine, University Hospital Münster, Albert-Schweitzer-Campus 1, Building D3, 48149 Münster, Germany. Tel: ++49 251 8357089 | Fax: ++49 251 8355747 |

E-Mail: rinesh.godfrey@ukmuenster.de

Prof. Dr. Johannes Waltenberger, MD, F.E.S.C., Professor and Chair of Internal Medicine, Cardiology and Vascular Medicine, Medical Faculty, University of Münster, Albert-Schweitzer-Campus 1 - Building A1, 48149 Münster, Germany. Tel: ++49 157 331 991 61 | Fax: ++49 3212 935 2935 | E-Mail: waltenberger@email.de

Supplementary Figure S1

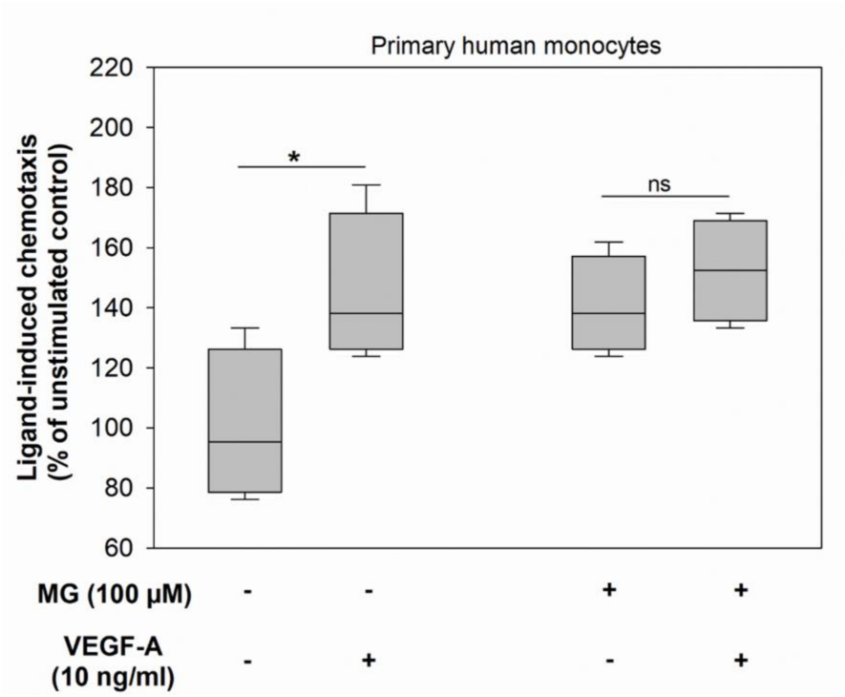

Supplementary Figure S1. Methlglyoxal impairs the ability of monocytes to respond to arteriogenic, VEGFR-1 activating ligand, VEGF-A.
Primary monocytes were exposed to 100 µM of MG for 24 hours. Cells were then serum starved and analysed for their migratory responses towards VEGF-A using Boyden chamber. The migrated cells were then fixed and stained with Giemsa and counted under a microscope. Kruskal-Wallis One Way Analysis of Variance on Ranks with Tukey correction was used for statistical analysis (*$P<0.05$).

Supplementary Figure S2

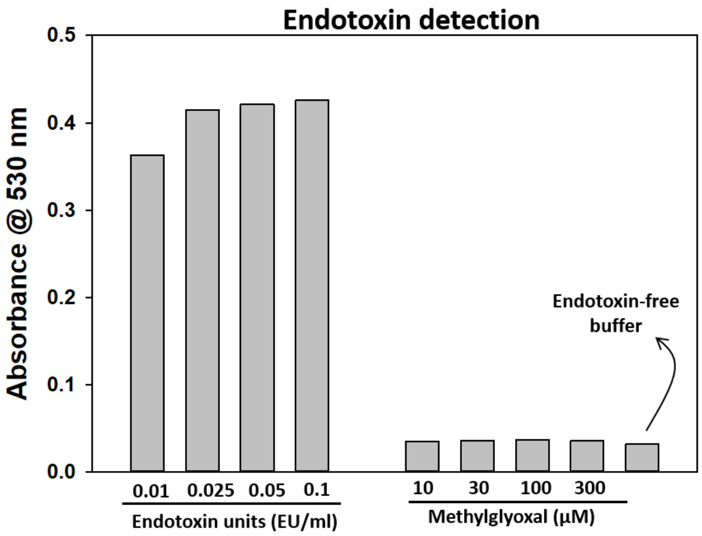

Supplementary Figure S2. Detection of endotoxin levels in different concentrations of methylglyoxal used for experiments.
Different concentrations of MG (10, 30, 100 and 300 µM) were tested for the presence of endotoxin using a modified Limulus Amebocyte Lysate (LAL) method. The final chromogenic product was detected at 530 nm using a spectrophotometer. Endotoxin-free buffer served as negative control.

Supplementary Figure S3

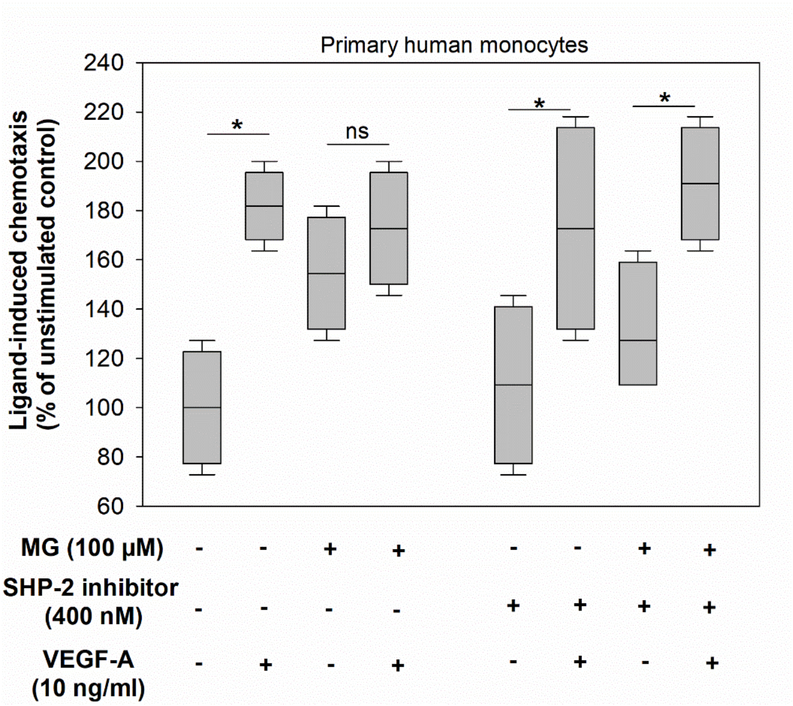

Supplementary Figure S3. Methlglyoxal-induced impaired chemotaxis towards VEGF-A is reversed by SHP-2 phosphatase inhibition.
Primary monocytes were exposed to 100 µM of MG for 24 hours with or without SHP-2 inhibitor (400 nM). Cells were then serum starved for 2 hours and analysed for their migratory responses towards VEGF-A using Boyden chamber. The migrated cells were then fixed and stained with Giemsa and counted under a microscope. Kruskal-Wallis One Way Analysis of Variance on Ranks with Tukey correction was used for statistical analysis (*P<0.05).

Supplementary Figure S4

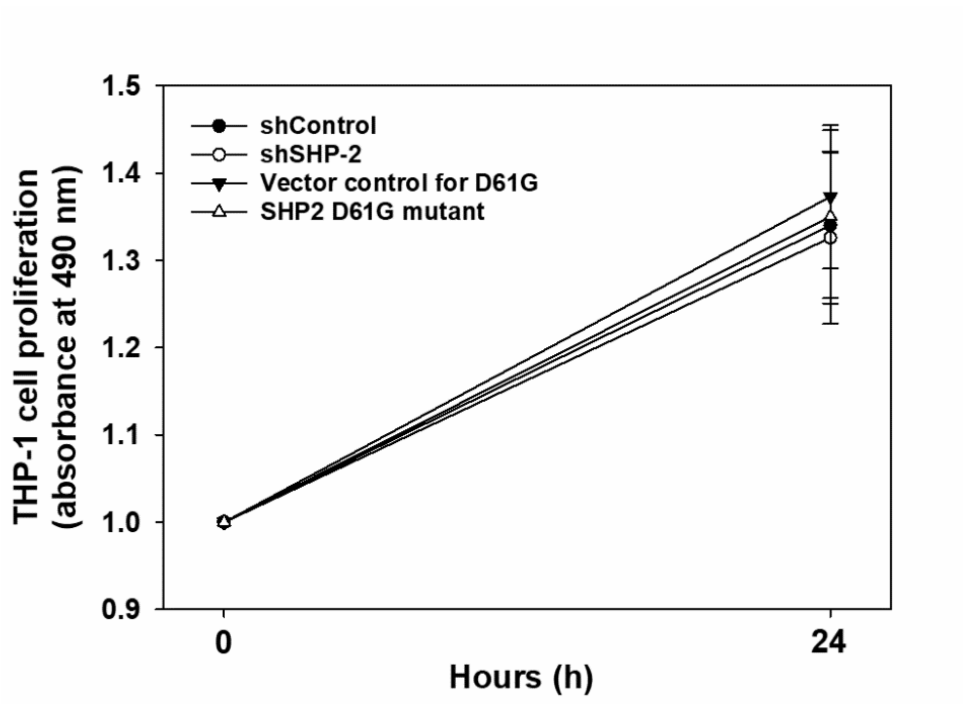

Supplementary Figure S4. Viability/proliferation rate of genetically manipulated THP-1 cells.

Stable THP-1 cells expressing the shControl, shSHP-2, vector control and D61G mutant were seeded at equal numbers (20,000 cells/well) in RPMI-1640 medium containing 10% serum and were allowed to proliferate for 24 hours. The bio-reduction of [3-(4,5-dimethylthiazol-2-yl)-5-(3-carboxymethoxyphenyl)-2-(4-sulfophenyl)-2H-tetrazolium (MTS) to formazan by metabolically active cells were measured. The quantity of formazan product as measured by the amount of 490nm absorbance is directly proportional to the number of living cells in culture. CellTiter 96® AQueous Non-Radioactive Cell Proliferation Assay (MTS) from Promega was used for the assay. Mann-Whitney Rank Sum Test was used for statistical analysis (P>0.05 was considered non-significant).

Supplementary Figure S5

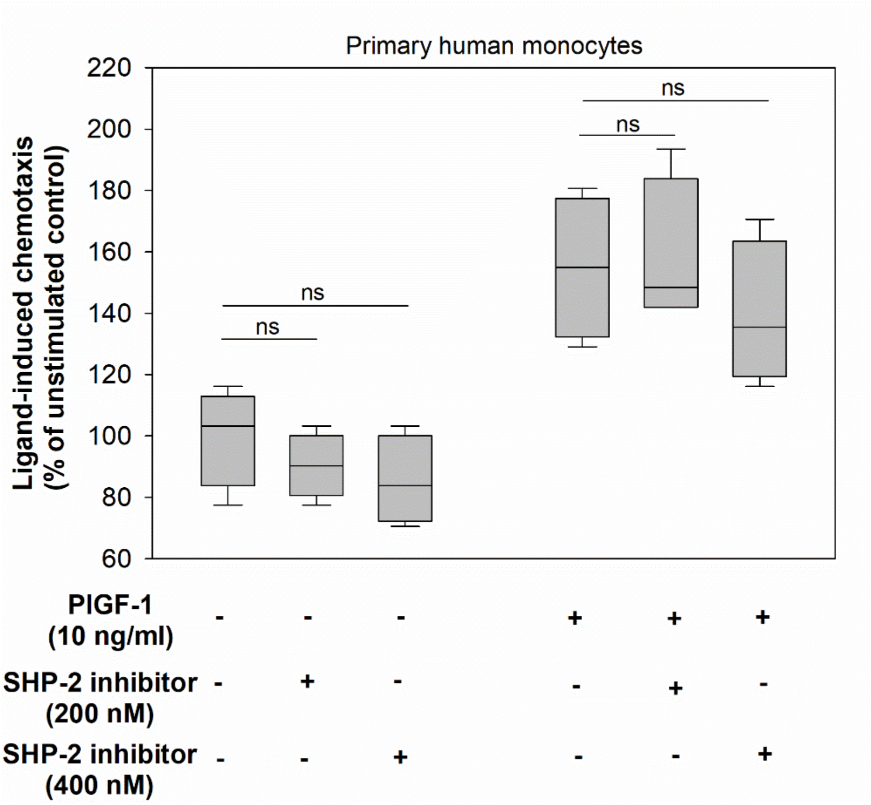

Supplementary Figure S5. Analysis of basal and PlGF-1-induced chemotaxis of monocytes treated with two different concentrations of SHP-2 inhibitor. Primary monocytes were exposed to 100 µM of MG for 24 hours with or without SHP-2 inhibitor at two different concentrations (200 nM and 400 nM). Cells were then serum starved for 2 hours and analysed for their migratory responses towards PlGF-1 using Boyden chamber. The migrated cells were then fixed and stained with Giemsa and counted under a microscope. Kruskal-Wallis One Way Analysis of Variance on Ranks with Tukey correction was used for statistical analysis (P>0.05 was considered non-significant).

Detailed description of experimental methods

Isolation of Primary Human and murine Monocytes

In brief, cells were provided by the blood bank of the University Hospital Muenster. 7.5 ml blood was transferred to 50 ml Falcon tubes. Equal amount of autoclaved phosphate buffered saline (PBS) was added. This mixture was carefully transferred to a Leucosep tube, containing 15 ml of Histopaque (Sigma Aldrich). Next, the tube was spun at 2200 rpm and room temperature (RT) for 20 minutes (min). Thereafter, concentrated mononuclear cells were taken to another 50 ml Falcon tube and mixed gently with 45 ml PBS. The tube was centrifuged for 10 min at 2200 rpm and RT. After centrifugation, the supernatant was removed, and 45 ml PBS was added to resuspend the pellet. Subsequently, tube was spun at 1200 rpm at RT for 10 min. The last two steps were repeated up to ten times to remove platelets. Afterwards, the reduction of platelet contamination was measured using CASY cell counter (Schärfe System). To isolate primary human monocytes, we used the Monocyte Isolation Kit II human from Miltenyi Biotec at RT according to manufacturer´s instructions.

Bone marrow cells from tibia and fibula were extracted by flushing with PBS-BSA solution. After erythrolysis, the cells were subjected to magnet-assisted cell sorting (MACS) according to the manufacturer's instructions. To isolate primary murine monocytes, we used the bone marrow monocyte isolation kit from Miltenyi Biotech at RT according to manufacturer´s instructions.

Cell culture

Primary human monocytes and the monocytic cell line THP-1 (acquired from Leibniz Institute DSMZ – German Collection of Microorganisms and Cell Cultures) were cultured in RPMI-1640 medium (+ L-Glutamine, - D-Glucose, Thermo Scientific) supplemented with 5

mM Glucose, 25 mM Mannitol, 10% heat-inactivated fetal bovine serum (FBS) and 1% Penicillin/Streptomycin. For migration experiments and signalling studies cells were starved for 2 hrs in FBS free medium. Monocytes were kept in an incubator at 37°C and 5% CO_2. Wherever indicated, cells were incubated with 400 nM SHP-2-inhibitor for 24 hours (hrs) or 100 nM SRC-inhibitor-1 for 6 hrs.

Monocyte chemotaxis and chemokinesis

Monocyte chemokinesis and chemotaxis were studied with the modified 48-well Boyden chamber (Nucleopore). 0.5×10^6 monocytes/ml were seeded in FBS free medium. Same medium without cells were filled in the wells of the lower part (28µl per well). Where indicated 10ng/ml of the attractant PLGF-1 was added. Subsequently, the lower part was overlayed with a 5µM nucleopore track-etched polycarbonate membrane for primary human monocytes or a 8µM nucleopore track-etched polycarbonate membrane for THP-1 cells. Afterwards, a silicon seal together with the upper part was connected to the lower part of the Boyden chamber with screws. Now each well of the upper part was filled with 50µl of the medium containing the monocytes. To allow migration of the cells towards the membrane, the Boyden chamber was placed in an incubator at 37°C and 5%CO_2 for 90 minutes. After incubation time screws were removed together with the upper part and the silicon seal. The lower side of the membrane held the migrated monocytes. Therefore, membrane was turned upside down and transferred into absolute ethanol for 10 minutes to fix the cells. Ethanol was washed away with H_2O and to stain the fixed monocytes membrane was transferred to Giemsa solution for 35 min. Membrane was washed again with H_2O and placed on a glass slide. To clean the lower part of the membrane from excessive Giemsa cotton swabs were used. Finally, by applying a mounting solution a cover-slide was attached on the lower side of

the membrane. For quantification migrated cells were counted by 20 high power fields in four different wells using the Axioskop 2 Plus microscope (Carl Zeiss).

Checkerboard assays were carried out as described above except that MG in FBS-free RPMI were placed in the top and in bottom wells of the Boyden chamber. In most of the experiments, unless indicated otherwise, cells were serum starved for 2 h prior to the assays.

SHP-2 Phosphatase activity measurement

In short, $8x10^6$ monocytes per sample were lysed in an anoxic chamber and a preclearing step was initiated by adding 20 µl protein G beads (Santa Cruz Biotechnology). Sample was continuously mixed on a rotating wheel at 12 rpm and 4°C for 1 hour. Afterwards, supernatant was pipetted into a new 1.5 ml Eppendorf tube. To perform immunoprecipitation, 5 µg SHP-2 antibody (Santa Cruz Biotechnology) and 30 µl protein G beads were added and sample was again placed on a rotating wheel at 12 rpm and 4°C for 2.5 hours. To measure the phosphatase activity reagents from the Tyrosine Phosphatase Assay System (Promega) were used. Absorbance was measured at 620 nm in the VICTOR X3 Multilabel Plate Reader (PerkinElmer).

SRC kinase activity measurement

In short, $10x10^6$ monocytes per sample were lysed and a preclearing step was initiated by adding 20 µl protein G beads (Santa Cruz Biotechnology). Sample was continuously mixed on a rotating wheel at 12 rpm and 4°C for 1 hour. Afterwards, supernatant was pipetted into a new 1.5 ml Eppendorf tube. To perform immunoprecipitation, 2 µg SRC antibody (Santa Cruz Biotechnology) and 30 µl protein G beads were added and sample was again placed on a

rotating wheel at 12 rpm and 4°C for 4 hours. To measure the kinase activity reagents from the Universal tyrosine kinase assay (Takara) were used. Absorbance was measured at 450 nm in the VICTOR X3 Multilabel Plate Reader (PerkinElmer).

Western Blot

Protein was isolated from cell pellet of seeded monocytes which was washed with 1ml ice-cold PBS and centrifuged at 1200 rpm and 4°C for 10 min. Cell pellet was resuspended in Radioimmunoprecipitation assay (RIPA) buffer, with phosphatase inhibitor and protease inhibitor (Thermo scientific). Protein concentration was determined by using the Bio-Rad Protein Assay Kit II according to manufacturer`s instructions, absorbance was measured at 700 nm in the VICTOR X3 Multilabel Plate Reader (PerkinElmer). Western Blot was performed as described in Tchaikovski *et al*[13]. PVDF-membrane was developed using ECL-substrate according to manufacturer´s instructions and picture was taken by Intelligent Dark Box LAS-3000 (FUJIFILM). Intensity of bands was quantified with ImageJ. Primary antibodies against SHP-2, SRC and Vinculin were from Santa Cruz Biotechnology, antibodies against Phospho-SRC (Tyr527), Non-phospho-SRC (Tyr527), SRC (32G6), phosphorylated paxillin (Y118) and total-paxillin were from Cell Signaling Technology. Secondary antibodies were from Santa Cruz Biotechnology.

DNA Expression Vectors, Production of Pseudoviral Particles and Cell Transduction

Plasmid pLKO.1 vectors encoding shRNA constructs targeting human SHP-2 and plasmid pLKO.1 encoding a nontargeting control shRNA were obtained from Sigma (MISSION® shRNA lentivirus-mediated transduction system, Taufkirchen, Germany).

The D61G mutant of SHP-2 was created using site-directed mutagenesis of an untagged SHP-2 cDNA and cloned into a retroviral vector.

For generation of retroviral particles, Phoenix amphotropic packaging cells (kindly provided by Dr. Gary Nolan, Stanford, CA) were maintained in Dulbecco's modified Eagle's medium (DMEM; Invitrogen) supplemented with 10% FCS. The cells were transiently transfected with the pMSCV-derivative plasmids using polyethyleneimine, and retrovirus-containing media were collected 48 h after transfection. 10^4 THP-1 cells were infected three times with the pseudotyped particles in the presence of 8 μg/ml Polybrene (1,5-dimethyl-1,5-diazaundecamethylene polymethobromide, Sigma). The transduction efficiency was in the range of 10–30% as assessed by parallel transduction with GFP-expressing viral particles. Selecting the population of the transduced cell pool with 2 μg/ml puromycin was started 48 h after transduction. For the creation of SHP-2-D61G cell line creation, THP-1 cells with a stable SHP-2 knockdown was used.

Production of lentiviral particles and transduction of THP-1 cells with corresponding cell supernatants was carried out essentially following the above protocol, except that Phoenix gp cells were used for virus particle production by transfecting them with pLKO.1 derivative plasmids in combination with pRev, pEnv-VSV-G, and pMDLg (kindly provided by Dr. Carol Stocking, Hamburg, Germany). Transduced cell pools were selected with 2 μg/ml puromycin. 10^4 THP-1 cells were infected three times with the pseudotyped particles in the presence of 8 μg/ml Polybrene. The transduced cell pool was selected with 2 μg/ml puromycin 48 h post-transduction and subjected to analysis. In each round of transduction, the corresponding control shRNA cell pools were generated and analyzed in parallel.

qPCR and primers used

RNA was isolated pursuant to the NucleoSpin RNA protocol "RNA purification from cultured cells and tissue" provided by MACHEREY-NAGEL. RNA-concentration was determined using the spectrophotometer NanoDrop ND-1000. From 2 µg of the isolated RNA cDNA was synthesized with cDNA synthesis kit (Thermo Scientific) according to manufacturer's instructions. The synthesized cDNA was used for Sybr Green (Thermo Scientific)-based qPCR reaction in CFX connect 96 Real-Time PCR Detection System (Biorad, Munich) with a 10 µl PCR mix that contained 2 µl of cDNA, 5 µl of SYBR Green PCR Master Mix (Thermo Scientific) and 300 nM of primer. The mix was preheated at 95°C for 15 min and then amplified in 40 cycles of 95°C for 30 sec, 60°C for 30 sec and 72°C for 30 sec. The threshold cycle (Ct) value of each sample was calculated and the expression of SHP-1 and SHP-2 mRNA relative to rplO or Hprt was determined by the 2−ΔΔCt method.

Connect Real-Time PCR Detection System (Bio-Rad Laboratories). The qPCR was done using the primers shown in Table. S1.

Table. S1: Sequences of the primers used in the study

Gene	Forward primer sequence (5' -3')	Reverse primer sequence (5' -3')
**hSHP-1**	ATGCAGAGACCCTGCTCAAG	ACCAGCTCTGTCAGAGTCG
**hSHP-2**	CCCACAATCAAGATTCAGAACACT	GCCCGTGATGTTCCATGTAA
**hrplO**	AATCTCCAGGGGCACCATT	CGCTGGCTCCCACTTTGT
**mSHP-1**	GCAAACTTTCCTGTCGCTGGTTGA	TTCGGGAGATATGGCACTACCAGT
**mSHP-2**	ACCCTGTGCAGAAATGAGGG	CCGTGGGCTCATCTGAAACT
**mHprt**	GCAGTACAGCCCCAAAATGG	AACAAAGTCTGGCCTGTATCCAA

Figure 2a – Antibody against SHP-2

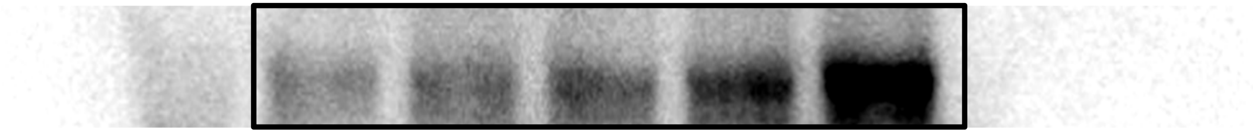

Figure 2a – Antibody against Vinculin

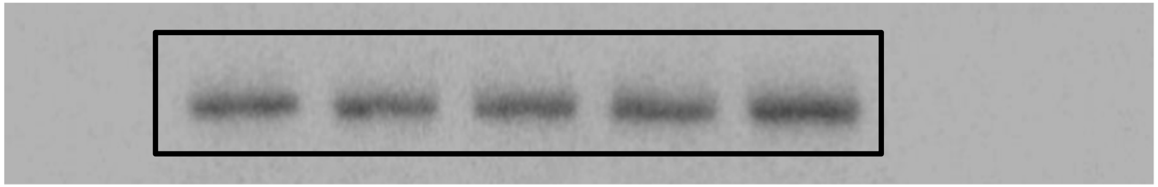

Figure 2e – Antibody against P-Tyr-527

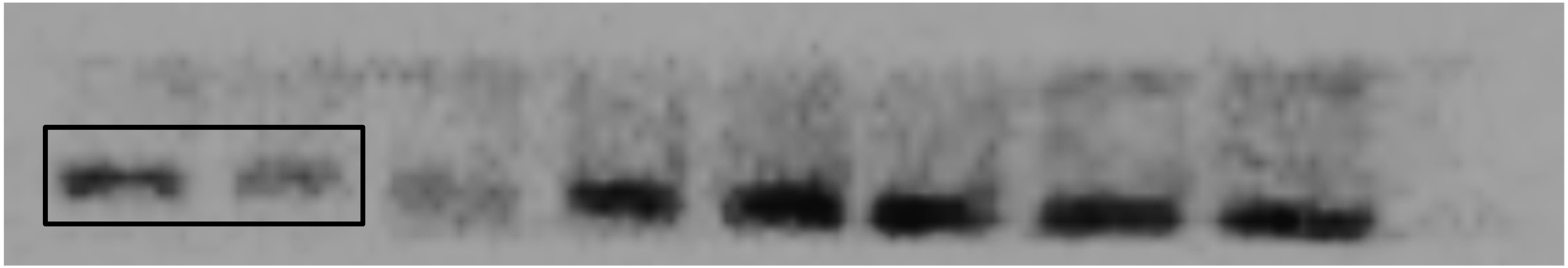

Figure 2e – Antibody against Src

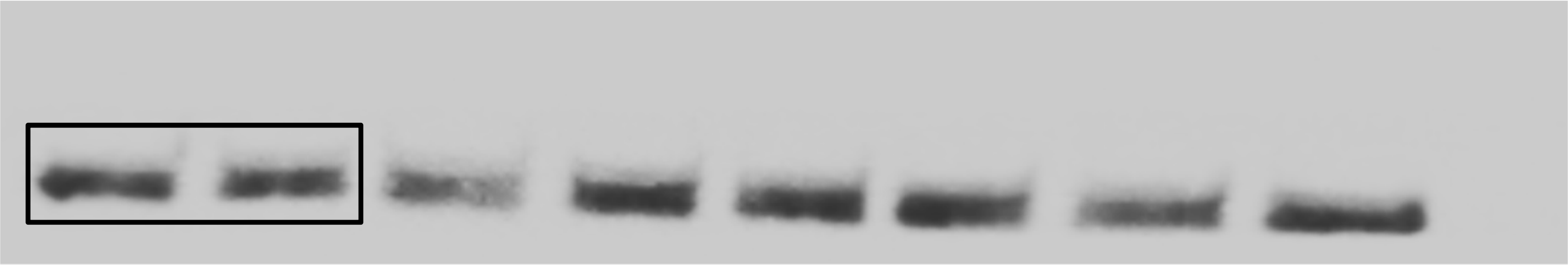

Figure 2g – Antibody against SHP-2

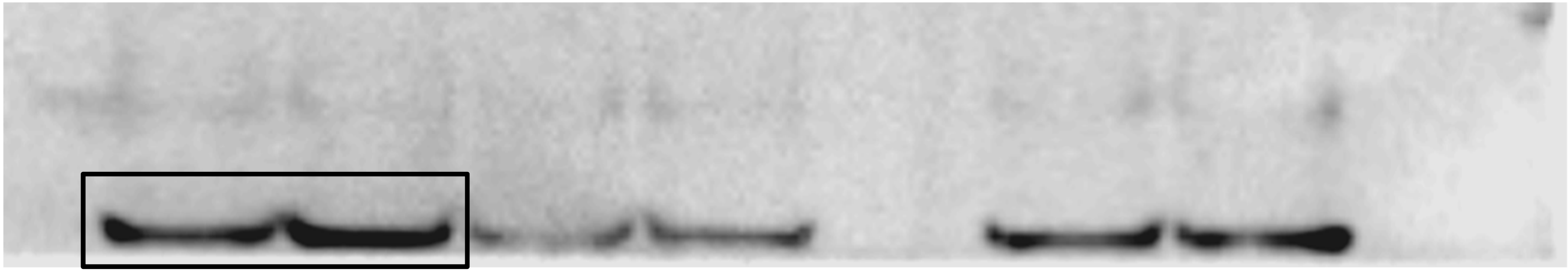

Figure 2g – Antibody against Src

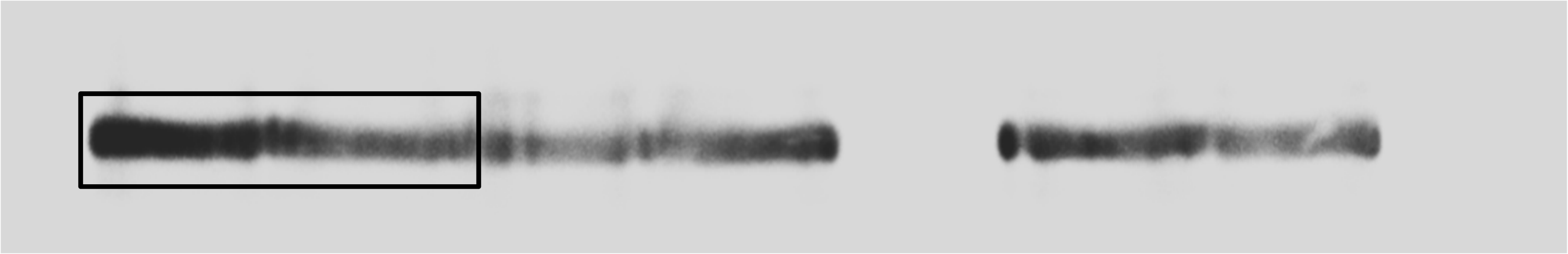

Figure 2i – Antibody against Src

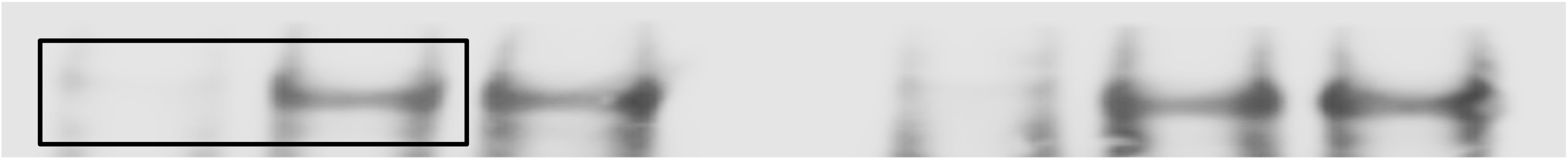

Figure 2i – Antibody against Vinculin

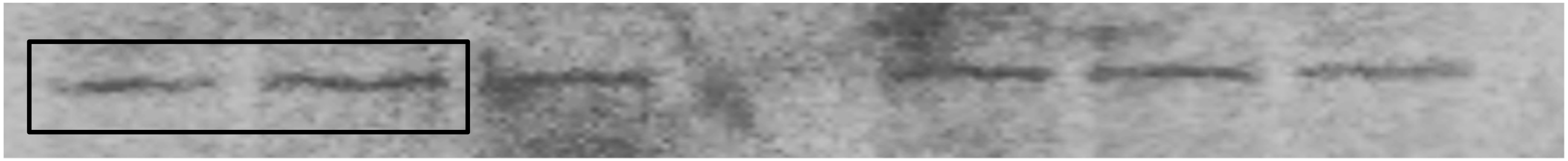

Figure 3e – Antibody against SHP-2

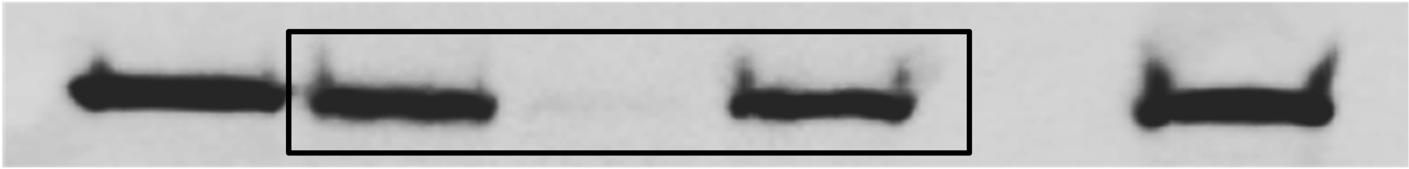

Figure 3e – Antibody against Vinculin

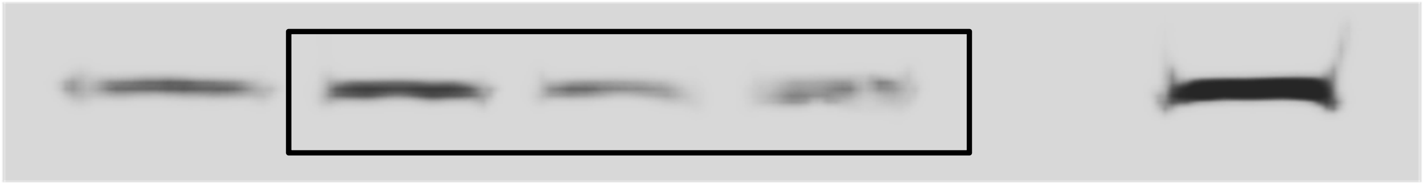

Figure 4a – Antibody against P-paxillin

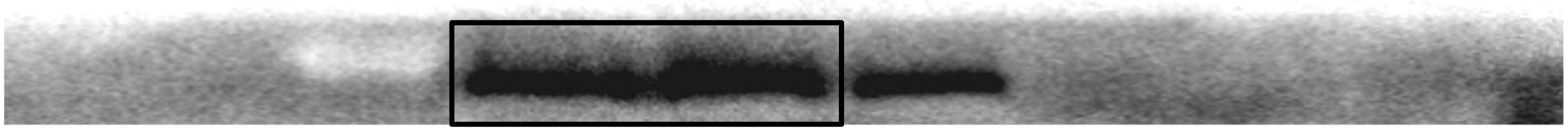

Figure 4a – Antibody against paxillin

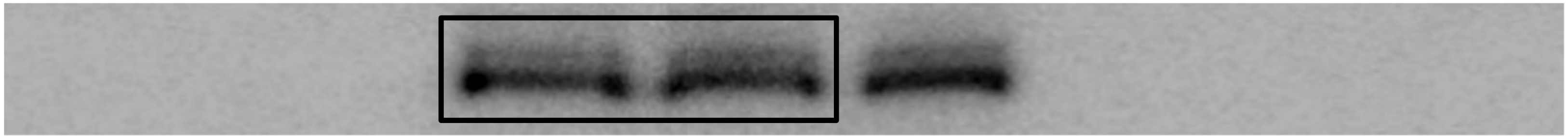

Figure 4c – Antibody against P-Tyr527

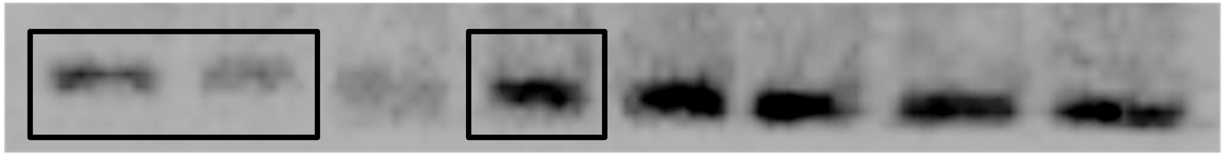

Figure 4c – Antibody against Src

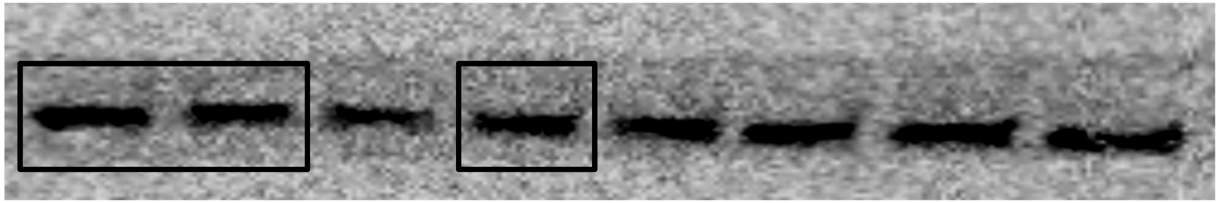

Blotted cropped from different parts of the same gel was used in Figure 4c. This has been explicitly mentioned in the figure legends. The full-length blots of the figure are shown above.

Figure 4e – Antibody against P-paxillin

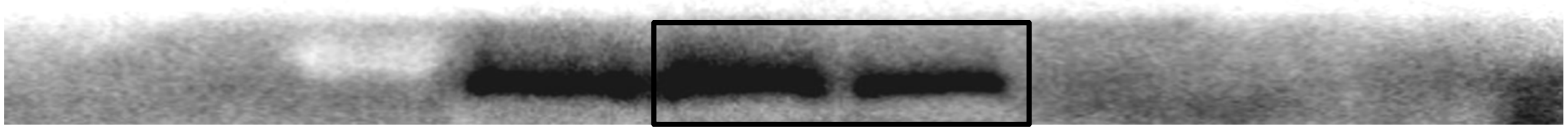

Figure 4e – Antibody against paxillin

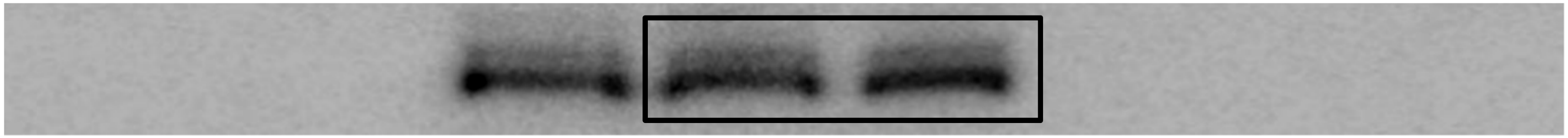

Figure 4g – Antibody against P-Tyr527

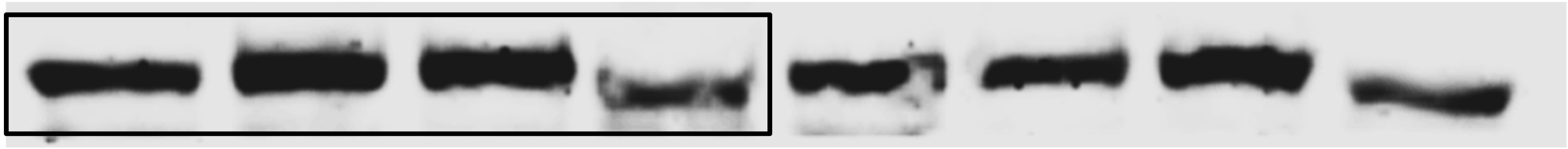

Figure 4g – Antibody against Tyr527

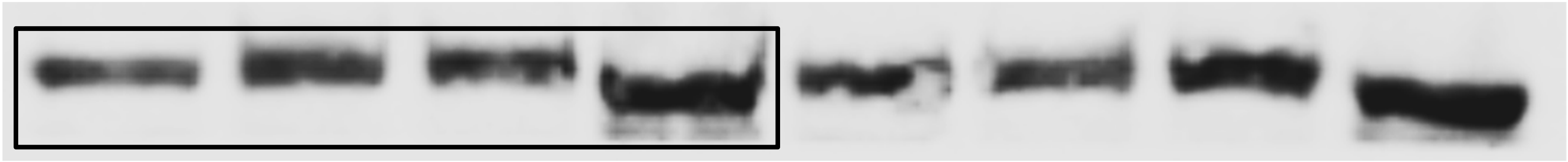

Figure 4i – Antibody against P-paxillin

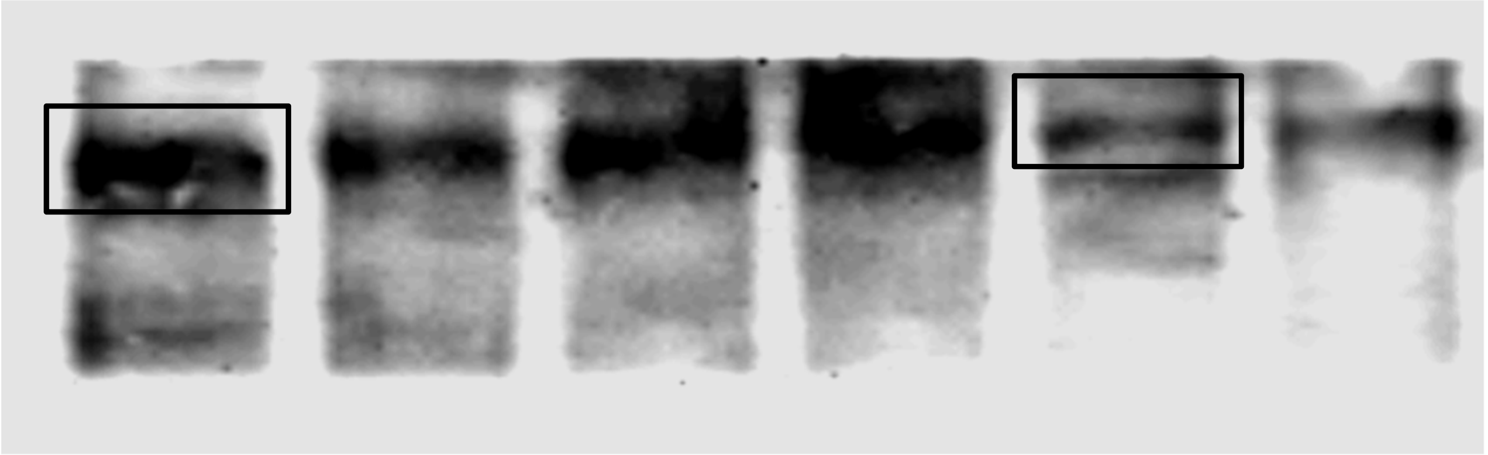

Figure 4i – Antibody against paxillin

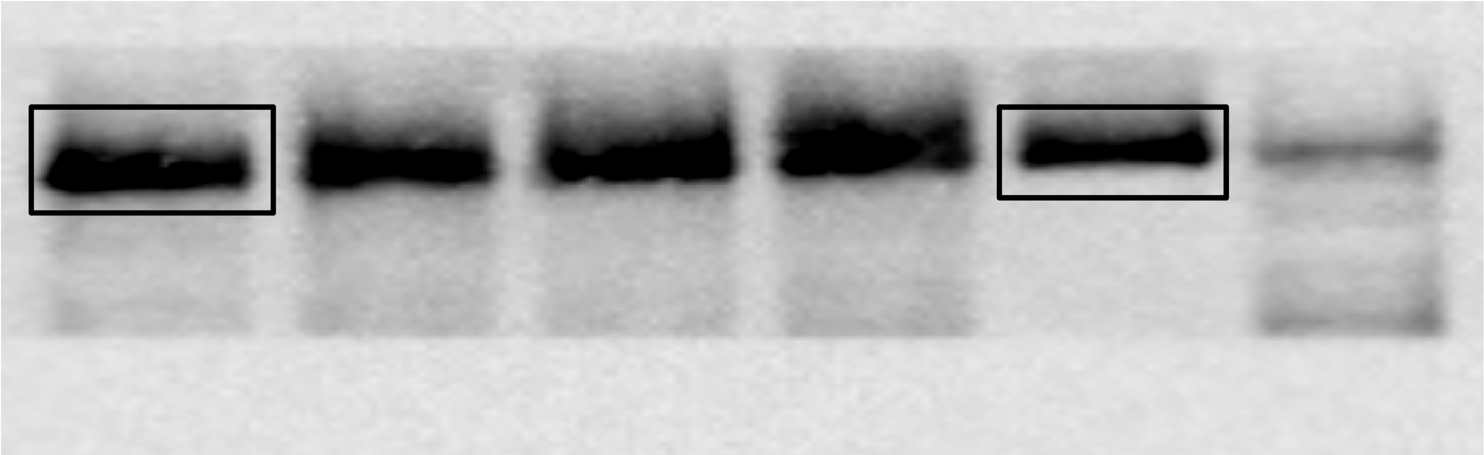

Blotted cropped from different parts of the same gel was used in Figure 4i. This has been explicitly mentioned in the figure legends. The full-length blots of the figure are shown above.

Figure 4k – Antibody against P-paxillin

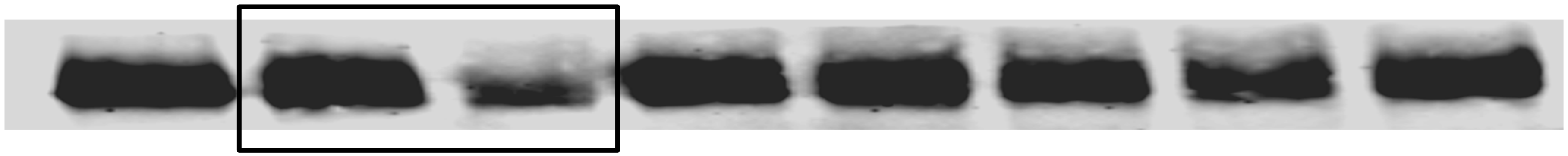

Figure 4k – Antibody against paxillin

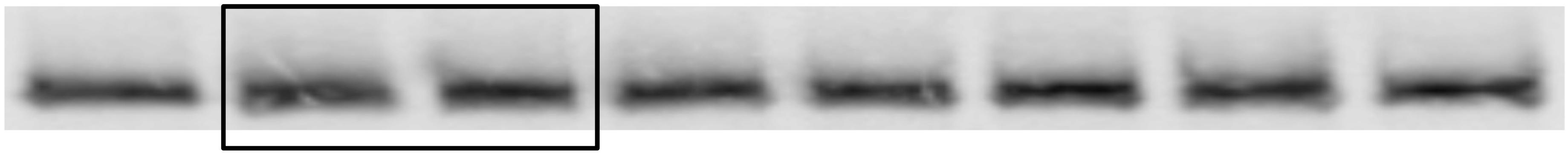

Supplement: Supplementary file 1 — Supplementary Information [file 41598_2018_33014_MOESM1_ESM.pdf]
